# Clinical and laboratory characteristics during a 1‐year follow‐up in European Lyme neuroborreliosis: A prospective cohort study

**DOI:** 10.1111/ene.16487

**Published:** 2024-09-19

**Authors:** Anne Marit Solheim, Ingerid Skarstein, Hanne Quarsten, Åslaug Rudjord Lorentzen, Pål Berg‐Hansen, Randi Eikeland, Harald Reiso, Åse Mygland, Unn Ljøstad

**Affiliations:** ^1^ Department of Neurology Sorlandet Hospital Kristiansand Norway; ^2^ Department of Clinical Medicine University of Bergen Bergen Norway; ^3^ Department of Microbiology Haukeland University Hospital Bergen Norway; ^4^ Department of Clinical Science University of Bergen Bergen Norway; ^5^ Department of Microbiology Sorlandet Hospital Kristiansand Norway; ^6^ Norwegian National Advisory Unit on Tick‐Borne Diseases Sorlandet Hospital Kristiansand Norway; ^7^ Department of Neurology Oslo University Hospital Oslo Norway; ^8^ Institute of Health and Nursing Science, University of Agder Kristiansand Norway; ^9^ Section for Adult Habilitation Sorlandet Hospital Kristiansand Norway

**Keywords:** biomarkers, case definition, clinical characteristics, neuroborreliosis, prognosis

## Abstract

**Background and Purpose:**

We need more knowledge on clinical presentations, time course, biomarkers, and prognosis in European Lyme neuroborreliosis (LNB).

**Methods:**

A prospective 12‐month follow‐up of predetermined clinical and laboratory parameters was undertaken in 105 patients with LNB.

**Results:**

At presentation, 79% had radiculopathy, 49% had facial palsy, and 13% had solely subjective symptoms (predominately pain). Intrathecally produced *Borrelia burgdorferi* (*Bb*) antibodies were demonstrated and cerebrospinal fluid (CSF) CXCL13 was positive in 85% and 82% pretreatment, in 73% and 10% at 6 months, and in 58% and 14% at 12 months, respectively. CSF *Bb* polymerase chain reaction (PCR) was positive in 40% pretreatment. In four patients who tested negative for *Bb* antibodies in both serum and CSF, the diagnosis was supported by typical clinical features, pleocytosis, CSF *Bb*‐PCR (*n* = 1), or CSF CXCL13 (*n* = 2). The proportion with symptoms influencing daily life was 91% pretreatment, 25% at 10 weeks, 20% at 6 months, and 15% at 12 months. Fatigue was the most common complaint at 12 months. A high burden of symptoms before and after treatment was associated with residual complaints at 12 months, whereas background data, other clinical features, and laboratory features were not.

**Conclusions:**

LNB can present with solely subjective symptoms, especially pain. Many LNB patients have persistent *Bb* antibodies in serum and CSF. In seronegative LNB, CSF *Bb‐*PCR and CXCL13 may give diagnostic support. CXCL13 may be persistently positive after treatment in some patients. Most of the clinical improvement occurs during the first 10 weeks. High initial clinical score is associated with poorer outcome.

## INTRODUCTION

European Lyme neuroborreliosis (LNB) is a tick‐borne disease in the nervous system caused by the bacterium *Borrelia burgdorferi* (*Bb*) sensu lato. Clinical presentation is predominantly of peripheral nervous system origin. Most typical are painful meningoradiculitis and/or cranial neuritis, often labeled Bannwarth syndrome. Parenchymal central nervous system (CNS) involvement causing encephalitis or myelitis is much less frequent [1]. Objective manifestations are generally accompanied by subjective symptoms such as fatigue, malaise, pain, and/or memory and concentration problems, whereas, according to current knowledge, LNB very rarely presents with exclusively subjective complaints [2].

Diagnosis of European LNB is based on a combination of typical clinical neurological presentation, pleocytosis, and/or *Bb* antibodies produced intrathecally (positive *Bb* antibody ratio). According to current guidelines [3], diagnosis of definite LNB requires all three criteria to be fulfilled, and possible LNB requires two of the three. The diagnostic process, however, may be challenging in clinical practice. First, the clinical presentations may be atypical or confusing; second, pleocytosis is unspecific and occurs in many conditions; and finally, a positive *Bb* antibody ratio may be absent in early phases of the disease and in immunosuppressed patients, and it may persist for years after a cleared LNB. Consequently, other laboratory diagnostic or supportive biomarkers are requested. Direct detection of the bacterium by polymerase chain reaction (PCR) has low diagnostic sensitivity (<25%) but approximately 100% diagnostic specificity [4, 5, 6] and can be helpful in early LNB and in patients with immunomodulatory treatments where antibody production may be inadequate [7]. CXCL13 is a chemokine produced by microglia to attract antibody‐producing B cells. It is measured in higher levels in cerebrospinal fluid (CSF) in acute LNB than in other neuroinflammatory diseases and may precede antibody production, and studies describe a rapid decline after initiated antibiotic treatment. CSF CXCL13 has therefore been proposed as both an early diagnostic marker and a marker of treatment response. Further research has been called for to verify this [8, 9, 10].

There is scant knowledge on the time course of recovery after antibiotic treatment, and both prevalence and underlying cause of long‐term residual complaints after LNB are debated. In a systematic review of 34 studies from 2015, 28% (23%–34%) of included patients had residual complaints after LNB [11]. The studies included, however, differed considerably regarding design, inclusion criteria, follow‐up times, and outcome measures. More recent studies found unfavorable outcome after treated LNB in 12%–45%, and significantly more complaints among patients classified with possible as compared to patients classified with definite LNB [2, 12]. The pathogenesis of residual complaints after treated LNB is not fully understood, but substantial evidence refutes the hypothesis of ongoing infection [13]. Other causes, such as pre‐existing cognitive–behavioral factors, negative illness perceptions, tissue damage, and immune responses, might be relevant; one prospective study found that cytokine and chemokine levels could be linked to LNB clinical features and outcome [9]. Another prospective study, primarily on patients with erythema migrans, found that the main predictors of outcome were baseline psychosociological characteristics [14]. Others have shown that delayed initiation of antibiotic treatment and a high burden of symptoms pretreatment are associated with reduced health‐related quality of life [15] and a higher burden of residual complaints [2, 16, 17, 18].

In summary, even if LNB is a well‐studied disease, we need more knowledge on the different clinical presentations, illness time course, biomarkers, and predictors of residual complaints. The objectives of our study were to assess the temporal course of clinical and laboratory findings in a well‐defined cohort of LNB patients and to identify possible prognostic factors.

## METHODS

### Study design

In this prospective cohort study, we selected patients from a multicenter treatment trial in South Norway on adult patients with definite and possible LNB according to the European Federation of Neurological Societies (EFNS) guidelines [19]. We excluded patients without CSF pleocytosis (defined as ≥5 cells/μL) and patients who turned out to have another diagnosis or with extensive missing data. Further details are found in a previous publication [20].

### Outcome measures

All patients were scored on a composite clinical score (CCS) pretreatment and at 10 weeks, 6 months, and 12 months after treatment. The CCS consists of 32 variables on subjective symptoms and objective findings related to the current LNB and was scored by an experienced physician based on patient history and a thorough clinical neurological examination. Subjective symptoms included newly emerged malaise, fatigue, headache, neck/back pain, localized pain in extremities or truncus, joint/muscle pain, and difficulties with memory/concentration. Each variable was scored from 0 to 2 points: 0 = none, 1 = mild symptoms without influence on daily life, and 2 = serious symptoms with influence on daily life. A poor long‐term outcome was defined as having one or more variables scored with 2 points on the CCS at 12 months. Based on a telephone call 1 week after starting treatment, symptom development was scored as 1 = increased, 2 = unchanged, 3 = improved a little, 4 = improved much, or 5 = no symptoms. Patient‐reported outcome measures (PROMs) were Fatigue Severity Scale (FSS) at all follow‐up times, Patient Health Questionnaire‐15 at 6 and 12 months, and RAND 36‐Item Short Form Health Survey (RAND‐36) at 6 months. CCS, study procedures, and PROMs are further described in previous publications and in Data S1.

### Laboratory data

*Bb* antibodies in serum and CSF, calculations of *Bb* antibody ratio, and oligoclonal bands (OCBs) restricted to CSF were analyzed in accordance with local procedures at each hospital. The presence of IgG and IgM antibodies was registered as positive or negative. Quantification of antibody levels and indexes were not available. CSF *Bb‐*PCR and CXCL13 analysis was performed retrospectively. DNA was isolated by MagNAPure 96 DNA or viral NA small volume kit (Roche, Mannheim, Germany) from 200‐μL CSF pellet materials. For analyzing CXCL13 in CSF, we used the kit *recom*Bead CXCL13 (Mikrogen Diagnostic, Neuried, Germany) and levels > 300 pg/mL were categorized as positive in accordance with the manufacturer's recommendation. More information on methods are provided in Data S2.

### Statistical analysis

Correlations between variables, as well as the endpoint of poor outcome, were evaluated using one‐way analysis of variance, *t*‐tests or Wilcoxon test for continuous variables, and Pearson chi‐squared and McNemar test for categorical variables; *p* < 0.05 was considered significant. Data were analyzed with the statistical program SPSS (SPSS IBM V.29).

### Ethics

The Norwegian Regional Committees for Medical and Health Research Ethics approved the treatment trial (reference 2015/1031) and the biobank (reference 2015/1033). The participants gave written informed consent before inclusion. The trial registration number is 2015‐001481‐25.

## RESULTS

**FIGURE 1:**
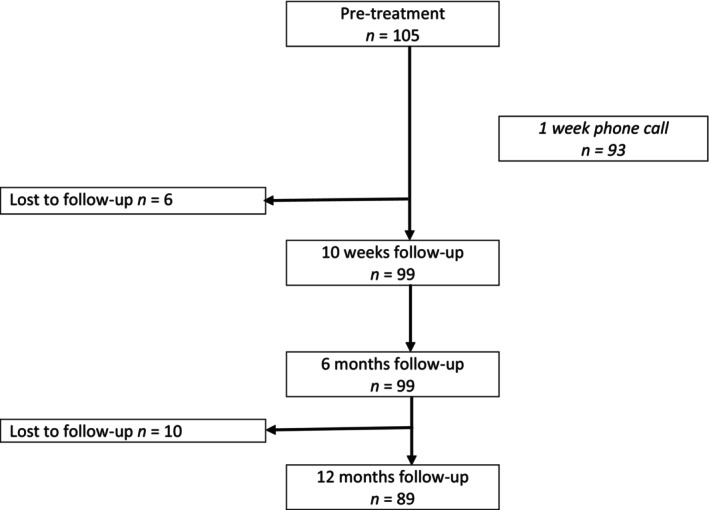
Diagram of the study population at the different times of follow‐up. Included patients from November 2015 to January 2020 from seven hospitals in South Norway are shown.

Figure 1 shows a flowchart of the 105 study participants included from November 2015 to January 2020, and Table 1 shows their baseline characteristics. Median pretreatment duration of symptoms was 21 days. Presence of facial palsy was associated with shorter pretreatment symptom duration (*p* = 0.010), whereas sex, age, living alone, and serious radicular pain or solely subjective symptoms did not influence time to treatment. None of the patients had ongoing erythema migrans.

**TABLE 1:** Background characteristics.

Characteristic	Value
*N*	105
Age, years, mean (SD)	56 (13)
Male	60 (57%)
Erythema migrans in past 6 months	21 (20%)
Tick bite in past 6 months	55 (52%)
Duration of symptoms, days, median (range)	21 (1–515)
Duration ≥ 42 days	16 (15%)
Living alone	26 (25%)
Education after elementary school	
≤3 years	42 (40%)
3–6 years	35 (33%)
>6 years	28 (27%)
Occupational status	
Full‐time job/part‐time job	62 (59%)
Age pensioner	29 (28%)
Unemployed/sick leave or disability full time	19 (18%)
Sick leave or disability part time	6 (6%)

*Note:* Data are given as *n* (%) unless otherwise stated.

At presentation, most patients had several symptoms and findings (Table 2). Two patients had solely facial palsy and one had solely radiculopathy (defined as radicular pain and/or hypoesthesia and/or paresis). Fourteen had solely subjective symptoms, of whom 12 had radicular pain (defined as newly emerged, localized pain in extremities or truncus), one had serious headache, and one had mild malaise and fatigue. In total, 70 patients (67%) had serious radicular pain and 34 (32%) were prescribed advanced pain medication (gabapentin, pregabalin, codeine, or opioid) for pain related to the LNB. One patient developed bilateral facial palsy within 1 week of treatment. Only one suffered from a defined CNS syndrome, with a magnetic resonance imaging‐confirmed cervical myelitis that resolved radiologically but left mild clinical sequelae.

**TABLE 2:** Clinical features and PROMs at different time points.

	Pretreatment, *n* = 105	10 weeks, *n* = 99	*p* ^a^	6 months, *n* = 99	*p* ^b^	12 months, *n* = 89	*p* ^c^
CCS							
Median (range)	9 (2–27)	3 (0–12)	*p* < 0.001	2 (0–12)	*p* = 0.08	1 (0–8)	*p* = 0.03
Total score = 0	0	24 (24)	‐	28 (28)	*p* = 0.45	20 (22)	*p* = 0.51
≥1 any variable scored 2	96 (91)	25 (25)	*p* < 0.001	20 (20)	*p* = 0.3	13 (15)	*p* = 0.4
≥1 objective variable scored 2	48 (46)	7 (7)	‐	5 (5)	‐	4 (5)	
Objective findings, median (range)	2 (0–9)	0 (0–6)	‐	0 (0–4)	‐	0 (0–4)	‐
Subjective symptoms, median (range)^d^	6 (0–18)	2 (0–9)	‐	1.5 (0–10)	‐	1 (0–7)	‐
Subjective symptoms only	14 (13)	29 (29)	‐	40 (40)	‐	41 (46)	‐
Clinical features							
Radiculopathy^e^	83 (79)	41 (41)	‐	35 (35)	‐	27 (30)	‐
Facial palsy	51 (49)	15 (15)	‐	9 (9)	‐	8 (9)	‐
Other cranial neuropathy							
Reduced hearing	1 (1)	2 (2)	‐	4 (4)	‐	3 (3)	‐
Eye muscle paresis	1 (1)	2 (2)	‐	0	‐	0	‐
Other^f^	11 (10)	2 (2)	‐	2 (2)	‐	1 (1)	‐
PROMs							
FSS							
Median (range)	5.3 (1–7)	3.4 (1–7)	*p* < 0.001	3.4 (1–7)	*p* = 0.14	3.3 (0–7)	*p* = 0.53
Score ≥ 4	72 (69)	45 (45)	*p* = 0.001	38 (38)	*p* = 0.23	32 (36)	*p* = 0.8
PHQ‐15							
Median (range)	‐	‐	‐	5 (0–19)	‐	4.5 (0–19)	*p* = 0.35
Score ≥ 10				19 (19)	‐	19 (21)	*p* = 0.78
RAND‐36							
PCS, mean (SD)	‐	‐	‐	47.4 (10.3)	‐	‐	‐
MCS, mean (SD)	‐	‐	‐	51.5 (9.1)	‐	‐	‐

*Note*: Data are given as *n* (%) unless otherwise stated [15, 21].

Abbreviations: CCS, composite clinical score; FSS, Fatigue Severity Scale; MCS, mental component summary; PCS, physical component summary; PHQ‐15, Patient Health Questionnaire; PROM, patient‐reported outcome measure; RAND‐36, RAND 36‐Short Form Health Survey.

^a^ Change in scores from pretreatment to 10 weeks.

^b^ Change in scores from 10 weeks to 6 months.

^c^ Change in scores from 6 months to 12 months. PHQ‐15 and RAND 36 did not differ from population data. FSS mean score and proportion of patients with serious fatigue (FSS ≥ 4) differed significantly from population data only at pretreatment and 10 weeks: *p* = 0.003 and *p* = 0.001 [1,2].

^d^ Subjective symptoms included malaise, fatigue, headache, neck/back pain, localized pain in truncus or extremities, joint or muscle pain, and subjective cognitive symptoms.

^e^ Radiculopathy defined as radicular pain localized to truncus or extremities and/or paresis and/or sensory findings.

^f^ Other includes trigeminal neuropathy and vestibular findings.

At inclusion, three patients had symptom duration > 6 months (range = 180–515 days), defined as late LNB [3]. All three had pleocytosis (18–224 cells) and positive *Bb* antibody ratio, and two had positive CSF *Bb*‐PCR and CXCL13. The total CCS varied from 3 to 14 points, and all had radicular pain with a clearly defined debut of symptoms. All improved after treatment, but none had CCS = 0 and one had a variable scored with 2 points (fatigue) at 12 months.

Laboratory results are shown in Table 3. Eighty‐six patients (85%) had definite and 19 had possible LNB pretreatment (all with pleocytosis, but negative [*n* = 15] or missing [*n* = 4] *Bb* antibody ratio). The patients with possible LNB are further described in Table 4. Three patients converted from negative antibody ratio pretreatment to positive at 6 months, and two had missing data pretreatment but positive antibody ratio at 6 months.

**TABLE 3:** Laboratory data at three times of follow‐up.

CSF	Pretreatment, *n* = 105	6 months, *n* = 69	12 months, *n* = 27
Cells/μL, median (range)	113 (7–752)	2 (0–28)	2 (1–5)
Protein, g/L, median (range)	1 (0.3–5.3)	0.4 (0.2–1)	0.4 (0.2–0.9)
Oligoclonal bands^a^			
≥2 bands	59/88 (67)	34/63 (54)	12/26 (46)
≥10 bands	16/88 (18)	10/63 (16)	3/26 (12)
*Bb* IgG positive	99/104 (95)	58/68 (85)	21/27 (78)
*Bb* IgM positive	71/104 (68)	17/68 (25)	5/27 (19)
*Bb* antibody ratio positive	86/101 (85)	49/67 (73)	15/26 (58)
CXCL13 positive	80/97 (82)	6/58 (10)	3/22 (14)
*Bb*‐PCR positive	39/99 (40)	‐	‐
Blood			
*Bb* IgG positive	97/103 (94)	71/91 (78)	58/78 (74)
*Bb* IgM positive	79/103 (77)	39/91 (43)	27/77 (35)
CRP, mg/L, median (range)	2 (0–28)	1 (0–14)	1 (0–52)
Leukocytes, ×10^9^/L, mean (SD)	7.2 (2.0)	6 (1.6)	6.1 (1.7)

*Note*: Data are given as *n* (%) unless otherwise stated.

Abbreviations: *Bb*, *Borrelia burgdorferi*; CRP, C‐reactive protein; CSF, cerebrospinal fluid; PCR, polymerase chain reaction.

^a^ Oligoclonal bands restricted to the CSF.

**TABLE 4:** Characteristics of the 19 patients with a negative CSF *Bb* antibody ratio pretreatment.

Patient	Pretreatment							10 weeks	6 months		
	Symptoms and findings	Symptom duration, days	CSF, cells/μL	CSF *Bb*‐PCR	CSF CXCL13	Serum *Bb* IgG	CCS total score	CCS total score	CCS total score	Serum *Bb* IgG	CSF *Bb* ratio
1	Serious subjective symptoms and facial palsy	1	287	N/A	N/A	pos	9	0	0	pos	pos
2	Mild headache, serious cranial neuropathy	7	44	neg	neg	pos	3	0	0	pos	pos
3	Serious subjective symptoms	35	100	neg	pos	pos	13	0	2	pos	pos
4	Serious radiculopathy and subjective symptoms	42	552	neg	pos	pos	12	4	6	pos	pos
5	Serious subjective symptoms, mild radiculopathy	10	29	pos	pos	pos	11	7	3	pos	pos
6	Serious fatigue and radiculopathy	21	53	pos	pos	pos	7	0	1	neg	N/A
7	Serious subjective symptoms and facial palsy	7	8	pos	pos	neg^a^	7	1	0	neg^a^	neg
8	Serious subjective symptoms, mild facial palsy	28	74	pos	pos	pos	11	0	0	pos	neg
9	Mild subjective symptoms, serious radiculopathy	10	86	neg	pos	pos	7	1	1	pos	neg
10	Serious fatigue and radiculopathy	11	43	neg	pos	neg^a^	12	3	3	neg^a^	N/A
11	Serious subjective symptoms and facial palsy	35	9	neg	pos	N/A	9	0	0	N/A	N/A
12^b^	Serious subjective symptoms, hearing loss, mild radiculopathy	12	169	neg	neg	pos	6	1	1	pos	neg
13^b^	Mild neck pain, serious facial palsy	2	7	neg	neg	neg^a^	3	N/A	0	neg^a^	neg
14^b^	Mild fatigue, serious radiculopathy	7	95	neg	neg	neg^a^	8	0	0	neg^a^	neg
15^b^	Serious fatigue and radiculopathy	30	16	neg	neg	pos	7	N/A	N/A	N/A	N/A
16^b^	Mild subjective symptoms, serious radiculopathy	21	82	neg	neg	pos	7	4	2	pos	neg
17^b^	Serious subjective symptoms, facial palsy, mild radiculopathy	14	32	N/A	N/A	neg	13	9	N/A	N/A	N/A
18^b^	Mild subjective symptoms, facial palsy, serious radiculopathy	2	37	neg	neg	pos	5	1	3	pos	N/A
19^b^	Serious radiculopathy	40	54	N/A	N/A	pos	11	0	0	pos	N/A

Abbreviations: *Bb*, *Borrelia burgdorferi*; CCS, composite clinical score; CSF, cerebrospinal fluid; N/A, not available; neg, negative; pos, positive.

^a^ Patients with persistently negative *Bb* IgG antibodies in serum.

^b^ Patients with negative CSF *Bb* antibody ratio at 6 months, negative CSF *Bb* polymerase chain reaction, and negative CSF CXCL13.

Four patients had no detectable *Bb* antibodies in serum or CSF throughout the study, of whom one had both positive CSF *Bb*‐PCR and positive CSF CXCL13 and one had positive CSF CXCL13. One additional patient had negative *Bb* antibodies in serum and CSF pretreatment, but follow‐up samples are lacking. The five all had had symptom duration ≤14 days and are further described in Table 4. One additional patient had negative antibodies in serum throughout the study, but intrathecally produced antibodies. This patient had 49‐day symptom duration, erythema migrans within the past 6 months, CSF cell count of 538 cells/μL, positive CSF *Bb‐*PCR, and positive CSF CXCL13.

CSF *Bb‐*PCR was positive in 40% (39/99) of the patients pretreatment. The PCR‐positive patients did not differ in symptom duration, CCS pretreatment, or outcome but had a higher level of pleocytosis (*p* = 0.05). Ninety‐nine patients were also tested for other tick‐borne pathogens in CSF (*Neoehrlichia mikurensis*, *Borrelia miyamotoi*, *Anaplasma phagocytophilum*, *Rickettsia spp*., *Bartonella spp*., *Francisella tularensis*, *Coxiella burnetii*, *Babesia* (*Bab*.) *divergens*, *Bab*. *venatorum*, and *Bab*. *microti*) with negative results. Eighty‐nine (85%) patients tested negative for herpes simplex virus and varicella zoster virus PCR in CSF. Seven of 86 (8%) patients had tick‐borne encephalitis (TBE) virus IgG antibodies in serum, and none had TBE virus IgM. TBE vaccination status was not registered.

At 6 months and 12 months, the *Bb* antibody ratio was persistently positive in 73% and 58% of the patients, serum *Bb* IgG was positive in 78% and 74%, and serum *Bb* IgM was positive in 43% and 35%, respectively. At 6 months, one patient had moderate pleocytosis (28 cells/μL) but no residual complaints or new symptoms, and 11 patients had low levels of pleocytosis (range = 5–8 cells/μL). At 6 and 12 months, 34 of 63 (54%) and 12 of 26 (46%) still had ≥2 OCBs, and 10 of 63 (16%) and three of 26 (12%) had ≥10 OCBs. Five patients had an increase in OCBs to ≥10 from pretreatment to 6 months.

CSF CXCL13 was persistently positive in six and three patients at 6 and 12 months. All of the CSF CXCL13‐positive patients at 6 months and two of the CSF CXCL13‐positive patients at 12 months were also positive pretreatment. The CSF CXCL13‐positive patients at 6 months had CSF cell counts ranging from 0 to 7 at this time point, and all had marked improvement on the CCS.

**FIGURE 2:**
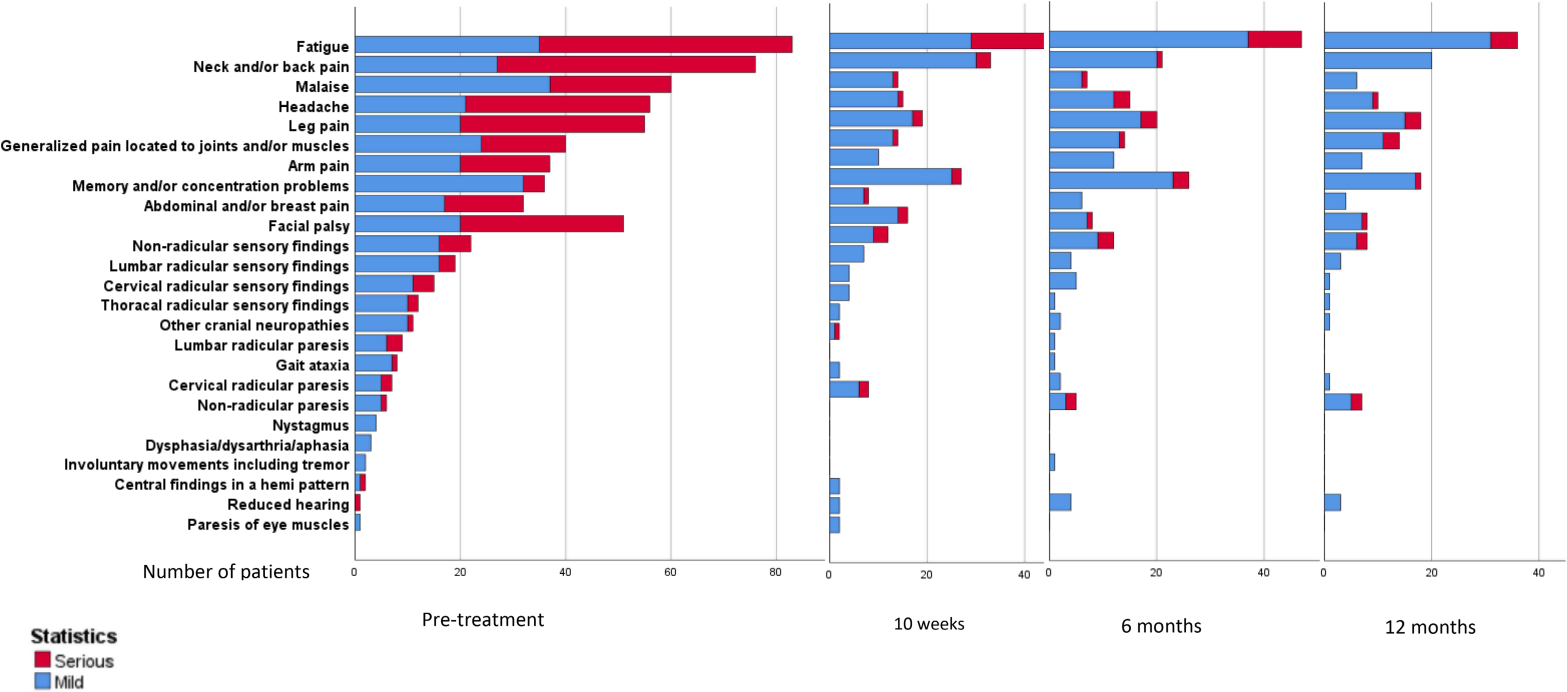
Symptoms and findings as scored on the composite clinical score variables at different times of follow‐up.

One week after start of treatment, two (2.2%) patients reported no symptoms, 71 (76.3%) improved symptoms, 15 (16.1%) unchanged symptoms, and five (5.4%) worsening of symptoms. The course of symptoms and findings during follow‐up are shown in Table 2 and Figure 2. The proportion of patients who scored 2 points on at least one variable on the CCS, indicating complaints with influence on daily life, was 25 of 97 (26%) at 10 weeks, 20 of 99 (20%) at 6 months, and 13 of 89 (15%) at 12 months.

Four patients received further oral antibiotics from their general practitioner outside of the treatment trial due to persisting symptoms after treatment. Experienced physicians and study personnel evaluated these patients and found no certain signs of treatment failure. One additional patient was treated for a suspected *Bb* arthritis that developed after the LNB.

CCS total score pretreatment (*p* = 0.02) and at 10 weeks (*p* < 0.001) and 6 months (p < 0.001), and FSS score pretreatment (*p* = 0.05) and at 10 weeks (*p* = 0.04) and 6 months (*p* = 0.002) were correlated with at least one variable scored with 2 points on the CCS at 12 months. No correlation was found for age, sex, education, living alone, pretreatment symptom duration ≥ 42 days, fulfilling criteria for definite LNB pretreatment, specific clinical syndromes (solely subjective symptoms, serious pain, radiculopathy, or facial palsy), CSF cell count, OCBs ≥ 2, and positive CSF CXCL13 and *Bb*‐PCR.

## DISCUSSION

In this prospective study of a well‐defined cohort of LNB patients with close clinical and laboratory follow‐up for 12 months, we made several interesting observations.

The predominant presenting symptoms and findings were fatigue, localized pain, malaise, radiculopathy, and facial palsy. Two patients had solely facial palsy without any accompanying symptoms, which could easily be confused with Bell's palsy. Thirteen patients with definite and one with possible LNB presented with solely newly emerged subjective symptoms: 12 with radicular pain and one with headache. In contrast, one previous study found no patients with definite LNB with solely subjective symptoms [2]. Others, however, describe a typical symptom debut with radicular pain, which is typically aggravated at night and is resistant to analgesics. There is a risk of diagnostic delay in such patients, as LNB is often only acknowledged at the debut of paresis [22]. Only one patient in our cohort had a confirmed CNS syndrome, which is fewer than previously reported [23]. A possible inclusion bias to the treatment trial was discussed in a previous publication [19].

The median duration of symptoms before treatment was 21 days. This is less than in previous studies, but the range was wide, and three patients had late LNB (defined as symptom duration for >6 months). All three improved after treatment. Facial palsy was associated with shorter symptom duration before treatment, whereas other clinical variables were not. This is in line with previous studies [12], and not surprising, as facial palsy is a conspicuous neurological finding known to be associated with LNB, and consequently prompts more urgent investigation than less defined symptoms such as pain. Our findings of a wide specter of clinical presentations and sometimes long pretreatment symptom duration support a low threshold for suspecting LNB and performing lumbar punctures in patients with newly emerged neurological symptoms and findings, especially in endemic areas. Particular awareness of LNB debut with pain and no initial objective findings is important to avoid delayed diagnosis.

The diagnostic EFNS criteria of “possible LNB” include patients either without pleocytosis or without positive *Bb* antibody ratio but require typical clinical presentation and exclusion of other causes. We did not include patients without pleocytosis in this cohort, but 19 had negative *Bb* antibody ratio pretreatment and were classified as possible LNB. In 11 of these patients, the diagnosis was laboratory supported by positive CSF *Bb‐*PCR (*n* = 4) and/or positive CSF CXCL13 pretreatment (*n* = 9), and/or positive *Bb* antibody ratio at 6 months (*n* = 5). PCR and CXCL13 results were not available at the time of diagnosis, as these analyses were performed later as a study procedure. We are confident in the LNB diagnosis of the remaining eight patients due to typical clinical presentations, improvement after treatment, pleocytosis, and exclusion of other plausible causes including coinfections. Furthermore, we did not see differing outcomes in the two diagnostic groups, in contrast to previous studies that have found a better outcome in definite LNB [2, 11, 12]. The contrasting results might reflect that the other studies included a high proportion of patients without pleocytosis who probably did not have LNB. Current knowledge of LNB pathophysiology implies that the bacteria elicit inflammation in the nervous system and thus CSF pleocytosis is a marker of ongoing infection [1]. Patients without pleocytosis should therefore be diagnosed with LNB with great caution.

Biomarkers with high diagnostic accuracy in LNB and the ability to discriminate between ongoing and previous disease have long been in demand. We found a positive *Bb* antibody ratio, CSF *Bb*‐PCR, and CSF CXCL13 in 85%, 40%, and 82%, respectively. At 6 and 12 months 73% and 58% were *Bb* antibody ratio positive, illustrating that this biomarker has limited specificity and is not suitable as a disease activity marker. Neither are serum *Bb* IgG and IgM, which were positive in 78% and 43% at 6 months, and 74% and 35% at 12 months. Nine of the 19 patients with a negative antibody ratio had positive CSF CXCL13 in the acute phase. They had similar clinical presentation and degree of pleocytosis as compared to patients with intrathecally produced antibodies but shorter duration of symptoms (mean = 22 days vs. 32 days), which is in line with previous studies [9]. The CXCL13 results would not have changed the treatment strategy, but still provided valuable diagnostic support. Surprisingly, we found that CSF CXCL13 was positive in 10% at 6 months and 14% at 12 months. This is in contrast with previous findings of rapid decline after treatment. As such, CSF CXCL13 is not optimal as a marker of disease activity. Our patients had high levels of CSF OCBs pretreatment and throughout the study as a sign of inflammation and immunological response. Interestingly, five patients had developed ≥10 OCBs at 6 months.

Whether persistent seronegative LNB exists is debated [6, 24, 25, 26]. Four of our patients were persistently *Bb* IgG antibody negative in serum and CSF throughout the follow‐up. They all had symptom duration ≤ 14 days, which might explain the absent antibodies. None had a medical history or medication to warrant a weakened immune response.

Our laboratory findings support further search for the ideal biomarker in LNB. Pleocytosis seems to be the best available activity marker, and both CSF *Bb*‐PCR and CXCL13 may give valuable diagnostic support. Seronegative patients with LNB seem to occur, and both IgM and IgG *Bb* serum antibodies, a positive *Bb* antibody ratio, and CSF‐exclusive OCBs may persist for >1 year after treated LNB.

Clinical improvement after LNB occurred early after the start of treatment. Already after 1 week, 76% reported symptom relief. Furthermore, the proportion of patients with at least one symptom influencing daily life was reduced from 91% before treatment to 26% at 10 weeks (*p* < 0.001), and the proportion with severe fatigue (FSS ≥ 4) was reduced from 69% to 46% (*p* = <0.001), whereas none of the proportions further improved after 10 weeks. The median CCS total score improved significantly from before treatment to 10 weeks, and between 6 and 12 months, but not between 10 weeks and 6 months. The proportion with no symptoms (total CCS = 0) increased from none to 25% at 10 weeks but did not increase after that. Clinical outcome 1 year after treatment among our patients was in line with earlier reports; 78% had any remaining complaint, 46% had solely subjective symptoms, 15% had any remaining complaint influencing daily life, and 5% had an objective complaint influencing daily life. PROM data on fatigue, somatic symptom load, and health‐related quality of life from follow‐up at 6 and 12 months did not differ from population data [21]. Our findings indicate that most LNB patients have a favorable prognosis overall, but some suffer from remaining complaints.

A high total symptom burden as measured by CCS and pronounced fatigue as measured by FSS score pretreatment and at 10 weeks and 6 months were associated with poor outcome at 12 months. A high symptom burden early in follow‐up being a negative prognostic factor is in line with a previous study with similar LNB case definition [18]. In contrast to previous studies, the outcome was not influenced by symptom duration before treatment, other clinical features, laboratory parameters, or LNB case definition [2, 12, 16, 17, 27]. Our patients had a shorter median pretreatment duration of symptoms compared to other studies, which might explain the contrasting results. Our findings indicate that a high burden of complaints before and after treatment is the most important negative prognostic factor.

Regarding the study's limitation, 16 patients were lost to follow‐up before 12 months, and a reporting bias toward patients with more residual complaints is possible. Additionally, there are no long‐term data on the patients treated with further antibiotics. Even so, the dropouts were few, and differed from the remaining patients only in age, not in initial clinical presentation [19]. Another possible limitation is the use of the unvalidated CCS, which had both intra‐ and interrater variability. Even so, the score reflects a thorough clinical assessment of these patients in daily practice, and the premise of use was specific scoring on symptoms and findings related to the current LNB only.

## CONCLUSIONS

LNB often presents with high symptom burden but sometimes with solely facial palsy or pain. Diagnostics can be complicated, and we recommend a liberal clinical practice of lumbar puncture in endemic areas. Our laboratory findings support further search for the ideal biomarker in LNB. Pleocytosis seems to be the best available activity marker, and both CSF *Bb‐*PCR and CSF CXCL13 may give valuable diagnostic support. Our results indicate that seronegative LNB exists, and that a considerable proportion of LNB patients have persistent serum IgM and IgG *Bb* antibodies, positive CSF *Bb* antibody ratio, and CSF‐exclusive OCBs for at least 1 year after treatment. Most of the recovery after treatment occurs within the first 10 weeks, but some improvement may occur later. One year after treatment, 15% had complaints influencing their lives. High initial burden of symptoms was associated with poorer outcome.

## AUTHOR CONTRIBUTIONS

**Anne Marit Solheim:** Investigation; writing – original draft; methodology; writing – review and editing; visualization; validation; software; formal analysis; data curation. **Ingerid Skarstein:** Data curation; writing – review and editing; investigation. **Hanne Quarsten:** Writing – review and editing; data curation. **Åslaug Rudjord Lorentzen:** Data curation; writing – review and editing; conceptualization; investigation; methodology; project administration; resources. **Pål Berg‐Hansen:** Investigation; writing – review and editing. **Randi Eikeland:** Conceptualization; investigation; writing – review and editing; methodology; project administration; resources. **Harald Reiso:** Conceptualization; validation; writing – review and editing; visualization; methodology; project administration; resources. **Åse Mygland:** Conceptualization; investigation; funding acquisition; writing – original draft; methodology; validation; visualization; writing – review and editing; formal analysis; project administration; data curation; supervision; software. **Unn Ljøstad:** Data curation; supervision; project administration; formal analysis; methodology; validation; visualization; writing – review and editing; writing – original draft; funding acquisition; investigation; conceptualization; software.

## CONFLICT OF INTEREST STATEMENT

The authors have no conflict of interest to declare.

## Supporting information


Data S1.

Data S2.

## Data Availability

The data that support the findings of this study are available from the corresponding author upon reasonable request. The data are not publicly available due to privacy or ethical restrictions.
