# Supplementary material for: Clinical and laboratory characteristics during a 1‐year follow‐up in European Lyme neuroborreliosis: A prospective cohort study
Source: Eur J Neurol. 2024 Sep 19;31(12):e16487. doi: 10.1111/ene.16487 (PMC11555137; doi:10.1111/ene.16487)
Supplement: Supplementary file 1 — Data S1. [file ENE-31-e16487-s002.docx]

Supplementary file on Composite clinical scale (CCS) and PROMS.

**Clinical composite score with 32 variables:**

| Subjective symptoms related by the patient to the current LNB |
| --- |
| -Malaise |
| -Fatigue |
| -Headache |
| -Neck and/or back pain |
| -Abdominal and/or breast pain |
| -Arm pain |
| -Leg pain |
| -Generalized pain located to joints and/or muscles |
| -Memory and/or concentration problems |
| -Other |
| Peripheral findings related to the current LNB |
| -Facial palsy |
| -Paresis of the eye muscles |
| -Reduced hearing |
| -Other cranial neuropathies |
| -Cervical radicular sensory findings |
| -Cervical radicular paresis |
| -Thoracic radicular sensory findings |
| -Lumbar radicular sensory findings |
| -Lumbar radicular paresis |
| -Non-radicular sensory findings |
| -Non-radicular paresis |
| -Other |
| Central findings related to the current LNB |
| -Central findings in one extremity |
| -Central findings in a hemi pattern |
| -Central findings in both legs |
| -Central findings in all extremities |
| -Gait ataxia |
| -Dysphasia/dysarthria/aphasia |
| -Nystagmus |
| -Involuntary movement including tremor |
| -Cognitive impairment |
| -Other |

Each point is scored from 0 to 2 points: 0 = none, 1 = mild symptoms without influence on daily life and 2 = serious symptoms with influence on daily life. Maximum 64 points. Radicular paresis or sensory findings follow the clinical pattern of a radicular nerve. Non-radicular paresis or sensory findings match a peripheral nerve or plexus.

**Fatigue severity scale (FSS):**

Comprised of nine items on the physical, social and cognitive effects of fatigue. Each item is scored on a Likert scale from 1 to 7, with 1= totally disagree and 7 = totally agree. The sum scores for the items are divided by nine. A high score indicates higher levels of fatigue. Internationally, mean scores ≥4 are considered a cut-off of severe fatigue. A cut-off of ≥5 is sometimes used in a Norwegian population.

**Patient Health Questionnaire (PHQ) - 15:**

Comprised of 15 items on somatic health where each is scored from 0 = not bothered at all, to 2= bothered a lot. The respondent is asked to only score symptoms from the last week. A cut off of ≥5 points represents low somatic symptom severity, ≥10 points represents medium severity and ≥15 points represent severe somatic symptoms.

**RAND 36-Item Short Form Health Survey:**

Comprises 36 health-related quality-of-life measures on an eight multi-item scale: Physical functioning, bodily pain, role limitations due to physical health problems or due to personal or emotional problems, general mental health, social functioning, fatigue and general health perceptions. The scales are standardized with a scoring algorithm to obtain scores ranging from 0 to 100. The summary scores are often reported separately in Physical component score (PCS) and Mental component score (MCS). Higher scores indicate better health status, and a mean score of 50 is generally used as a normative value.
