# Supplementary material for: Clinical and laboratory characteristics during a 1‐year follow‐up in European Lyme neuroborreliosis: A prospective cohort study
Source: Eur J Neurol. 2024 Sep 19;31(12):e16487. doi: 10.1111/ene.16487 (PMC11555137; doi:10.1111/ene.16487)
Supplement: Supplementary file 2 — Data S2. [file ENE-31-e16487-s001.docx]

Laboratory method:

CSF samples of 0.3-9 ml were centrifuged at 2300X g for ten min. All the pelleted material was resuspended in 200µl CSF. The CSF pellet material and 500µl aliquots of CSF were stored at -70°C.

CSF PCR

DNA was isolated by MagNAPure 96 DNA or viral NA small volume kit (Roche, Mannheim, Germany) from 200µl CSF pellet materials. DNA samples was tested by real-time PCR for detection of *Borrelia burgdorferi (Bb)* (two assays targeting the *ospA* and 16S rRNA genes)*, Borrelia miyamotoi*, *Anaplasma phagocytophilum*, *Rickettsia* spp.*,* *Neoehrlichia mikurensis* (assay designated CNM-II in reference), *Babesia microti*, *Babesia divergens*, *Babesia venatorum* as previously published.(1) PCR assays for detection of *Bartonella* spp*., Coxiella burnetii* and *Francisella tularensis* were perform as described.(2) DNA samples were tested once by all assays, except for the two Bb assays where they were tested in duplicates. Samples with inconsistent results (positive in one out of four reactions) in the duplicate runs were subsequently analysed in triplicate. Any positive reaction in the triplicate runs confirmed result from duplicate runs as positive.

CXCL13

For analyzing CXCL13 in CSF, all samples were retrospectively processed at University of Bergen, using the kit recomBead CXCL13 (Mikrogen diagnostik, Neuried, Germany) on a Luminex100 instrument. The assay contains fluorescence-marked beads covered with CXCL13-specific antibodies. CXCL13 in CSF will eventually bind to the antibodies, and, after addition of conjugate, this antibody-antigen-complex is detected by fluorescence. The CXCL13-levels was calculated, and levels above 300 pg/mL was categorized as positive in accordance with manufacturer’s recommendation. The spinal fluids were not freeze-tawn after biobanking before this analysis.

1. Quarsten H, Salte T, Lorentzen AR, Hansen IJW, Hamre R, Forselv KJN, et al. Tick-borne Pathogens Detected in the Blood of Immunosuppressed Norwegian Patients Living in a Tick-endemic Area. Clinical infectious diseases : an official publication of the Infectious Diseases Society of America. 2021;73(7):e2364-e71.

2. Quarsten H, Skarpaas T, Fajs L, Noraas S, Kjelland V. Tick-borne bacteria in Ixodes ricinus collected in southern Norway evaluated by a commercial kit and established real-time PCR protocols. Ticks and tick-borne diseases. 2015;6(4):538-44.
